# TRADITIONAL NETWORK ON MENTAL HEALTH: SUPPORT FROM CHILDREN AND DEPRESSIVE SYMPTOMS FOR OLDER ADULTS IN RURAL CHINA

**DOI:** 10.1093/geroni/igz038.1889

**Published:** 2019-11-08

**Authors:** Lei Chen, Fernando Torres-Gil

**Affiliations:** 1 Department of Social Welfare, Luskin School of Public Affairs, University of California, Los Angeles (UCLA), Los Angeles, California, United States; 2 Department of Social welfare and Public Policy, Luskin School of Public Affairs, University of California, Los Angeles, Los Angeles, California, United States

## Abstract

In rural China, support from children is the traditional network for older adults. Their mental health problem is poorly understood and remains unsolved. The study aims to explore the relationships between older adults’ depressive symptoms and characteristics of support from children under the current social-structural conditions in rural China. The study is informed by the intergenerational solidarity theory and the theoretical framework of social relationships and their influence on health. The quantitative study is based on the recent wave of Harmonized China Health and Retirement Longitudinal Study (Harmonized CHARLS) in 2015, which is a high-quality national-level dataset. The study applies conditional process analysis to do the data analysis. The key findings include: the number of children has a negative association with older adults’ depressive symptoms (c= -.2390, p=.0002<0.05); the number of children influenced older adults’ depressive symptoms indirectly through financial support from children (If children do not live with older parents, ab=-.0141, CI= -.393 to -.0038; If children live with older parents, ab=-.0153, CI= -.369 to -.0056;). However, both theses direct and indirect relationships do not depend on the co-residence situation between older adults and their children. The controlling variables include age, gender, and self-rated health. Under China’s current transition period of population policy, this study provides policy implications regards to the characteristics of children support and their influence on older adults’ mental health, especially in rural China. This study also tests the two theories to some extent under the Chinese context, which were initially developed in Western countries.

